# Supplementary material for: Emphasis Learning, Features Repetition in Width Instead of Length to Improve Classification Performance: Case Study—Alzheimer’s Disease Diagnosis
Source: Sensors (Basel). 2020 Feb 10;20(3):941. doi: 10.3390/s20030941 (PMC7039233; doi:10.3390/s20030941)
Supplement: Supplementary file 1 [file sensors-20-00941-s001.pdf]

Supplementary Material

After reviewing the manuscript, we realized that we did not consider the importance of features. Then, for more comparisons in terms of selecting features and repeating them, we prepared complementary materials. It is possible to repeat features using other strategies like weighting features, selecting top 10 PCs etc.

After we obtain features using PCA, they can be chosen one of two repetition strategy; that is, Homogeneous and Heterogeneous.

Homogeneous Emphasis Learning: Repeating all the obtained features without considering importance of them.

Heterogeneous Emphasis Learning: Repeating obtained features considering importance of them. For example, variance of PCs can be considered.

The new hypothesis was, if we selected top 10 high variance principal components and apply our method, the results might be better. Based on this hypothesis, we have conducted eight new experiments which revealed that our previous hypothesis that used no bias in selection worked better.

Here, the performance of both the Homogeneous and Heterogeneous Emphasis Learning methods are represented. The Homogeneous Emphasis Learning achieves best performance. However, the Heterogeneous method achieves slightly lower performance that is acceptable. In the Homogeneous Emphasis Learning, we repeat all the selected features equally and homogeneously, and in the Heterogeneous Emphasis Learning, we repeat selected features unequally and heterogeneously.

Homogeneous Emphasis part in the Table 1 shows the performance of applying the Homogeneous Emphasis Learning, and the Heterogeneous Emphasis part of the table represents the Heterogeneous Emphasis Learning. The 10th row represent the performance of the heterogeneous repetition using 25 PCs, which includes more than 97 percent of the variance, plus the first 15 PCs with 10 times repetition. Here, the accuracy is 1% less than Homogeneous Emphasis Learning with 5 times repetition. The 11th row represent the performance of the heterogeneous repetition using 25 PCs, plus the first 10 PCs with 10 times repetition. Here, the accuracy is 1% less than Homogeneous Emphasis Learning with 5 times repetition, too. The 16th row shows the performance of heterogeneous repetition using 25 PCs in addition to 8 first PCs with 5 times repetition, 8 second PCs with 4 times repetition, and next 9 PCs with 3 times repetition. Here, also, the accuracy is 0.2 % less than Homogeneous Emphasis Learning with 5 times repetition. The outcome of heterogeneous Emphasis Learning using 25 PCs with Weighted Approach applied to the PCs is shown in 17th row. Here, there was a high decrease in accuracy comparing to the Homogeneous Emphasis Learning with 5 times repetition. To employ this approach, number of repetition of each PC was equal to round value of the variance of each PC multiplied by a particular coefficient. This approach, has been tested with different coefficients

and did not yield much different results; the presented results are computed with a coefficient of 1.5.

Table 1. Comparing performance metrics. Classification accuracy (ACC), sensitivity (SEN), specificity (SPE), positive predictive value (PPV), negative predictive value (NPV), and area under the curve (AUC) for all features and 25 principal component analysis (PCA) elements.

	Data	Classes	ACC (%)	SEN (%)	SPE (%)	PPV (%)	NPV (%)	AUC
All	All Data	AD–NC	95.54	93.74	98.32	98.84	91.09	0.9577
		AD–MCI	81.41	89.02	68.09	82.99	78.00	0.7835
		MCI–NC	79.41	67.48	92.37	90.56	72.34	0.7993
PCA	Reduced Data Using PCA	AD–NC	97.20	95.46	**99.86**	**99.90**	93.53	0.9768
		AD–MCI	**81.61**	88.45	**69.02**	**84.03**	76.39	**0.7846**
		MCI–NC	79.45	67.20	**92.96**	**91.33**	71.97	0.8011
Homogeneous Emphasis	2 × Reduced Data	AD–NC	98.03	97.18	99.26	99.47	96.09	0.9831
		AD–MCI	80.37	88.57	66.38	81.80	77.28	0.7766
		MCI–NC	79.94	68.49	91.64	89.31	74.03	0.7991
	3 × Reduced Data	AD–NC	98.61	98.15	99.27	99.47	97.46	0.9863
		AD–MCI	80.47	88.90	66.26	81.62	77.98	0.7767
		MCI–NC	79.93	68.70	90.80	87.85	74.98	0.7980
	4 × Reduced Data	AD–NC	98.67	98.24	99.27	99.47	97.59	**0.9876**
		AD–MCI	80.61	88.92	66.59	81.81	78.02	0.7784
		MCI–NC	80.55	69.84	90.62	87.47	76.20	0.7998
	5 × Reduced Data	AD–NC	**98.81**	98.52	99.21	99.42	97.98	**0.9875**
		AD–MCI	80.69	89.46	66.37	81.29	79.39	0.7803
		MCI–NC	80.92	70.64	90.17	86.60	77.36	0.8016
	6 × Reduced Data	AD–NC	98.59	98.51	98.69	99.03	97.98	0.9866
		AD–MCI	80.81	89.47	66.65	81.46	79.43	0.7793
		MCI–NC	80.67	70.26	90.05	86.43	77.05	0.8045
	7 × Reduced Data	AD–NC	98.50	**98.61**	98.37	98.80	**98.11**	0.9852
		AD–MCI	80.71	89.07	66.66	81.82	78.32	0.7778
		MCI–NC	**81.44**	71.28	90.54	87.09	77.89	0.8056
	8 × Reduced Data	AD–NC	98.34	98.56	98.04	98.56	98.04	0.9835
		AD–MCI	80.84	**89.55**	66.57	81.45	**79.52**	0.7789
		MCI–NC	81.42	**71.51**	90.30	86.84	**77.98**	0.8075
	9 × Reduced Data	AD–NC	98.31	98.41	98.18	98.65	97.85	0.9822
		AD–MCI	80.51	88.91	66.43	81.62	78.13	0.7767
		MCI–NC	81.40	71.22	90.54	87.09	77.82	**0.808**
Heterogeneous Emphasis	Reduced Data + 10x15 PCs	AD–NC	97.89	97.17	98.92	99.23	96.09	0.9822
		AD–MCI	79.61	87.76	65.57	81.49	75.60	0.7648
		MCI–NC	79.33	67.68	91.28	88.83	73.38	0.7958
	Reduced Data + 10x10 PCs	AD–NC	97.89	97.09	99.05	99.33	95.94	0.9789
		AD–MCI	81.12	88.69	67.85	82.88	77.35	0.7820
		MCI–NC	79.15	67.29	91.79	89.73	72.47	0.7973
	Reduced Data + 5x10 PCs	**AD–NC**	**97.87**	**96.90**	**99.26**	**99.47**	**95.71**	**0.9868**
		**AD–MCI**	**81.04**	**88.58**	**67.66**	**82.95**	**76.91**	**0.7793**
		**MCI–NC**	**79.33**	**67.49**	**91.86**	**89.76**	**72.77**	**0.8007**
	Reduced Data + 3x10 PCs	**AD–NC**	**97.89**	**96.81**	**99.46**	**99.61**	**95.58**	**0.9122**
		**AD–MCI**	**81.49**	**88.76**	**68.49**	**83.43**	**77.31**	**0.7066**
		**MCI–NC**	**80.13**	**69.77**	**89.49**	**85.69**	**76.63**	**0.7138**

	**Reduced Data + 10x25 PCs**	**AD–NC**	**97.98**	**98.17**	**97.72**	**98.31**	**97.53**	**0.9780**
		**AD–MCI**	**79.41**	**88.28**	**64.84**	**80.50**	**77.08**	**0.7693**
		**MCI–NC**	**80.46**	**70.37**	**89.29**	**85.18**	**77.51**	**0.7971**
	**Reduced Data + 5x25 PCs**	**AD–NC**	**98.34**	**98.28**	**98.42**	**98.84**	**97.66**	**0.9821**
		**AD–MCI**	**79.53**	**88.16**	**65.10**	**80.87**	**76.65**	**0.7757**
		**MCI–NC**	**80.55**	**70.63**	**89.28**	**85.25**	**77.59**	**0.7972**
	**Reduced Data + 5x8 1stPCs + 4x8 2ndPCs +3x9 3rdPCs**	AD–NC	98.70	98.56	98.88	99.18	98.05	0.9870
		AD–MCI	80.33	88.88	66.01	81.41	77.99	0.7793
		MCI–NC	80.78	70.41	90.29	86.93	76.89	0.8010
	**Reduced Data + Weighted using variance x 1.5 and other factors**	AD–NC	90.80	91.21	90.23	92.97	87.88	0.9086
		AD–MCI	72.59	86.09	54.95	71.41	75.13	0.7031
		MCI–NC	70.54	59.00	82.89	78.69	65.38	0.7096

Increasing the dimensions (even repeating them) results in changes regarding the boundaries of decision (margins and hyper-planes) for SVM. SVM changes its boundaries of decision through dimension increase (Kernel Trick) too. SVM do not recognize whether the dimensions are repetitive; and with change of this size in dimensions, recognizing the equality of some dimensions for SVM is not as simple as a mathematic calculation. In this stage, the only thing we have to do is to find the best combination of features for emphasis and to create the highest performance, the results of which is represented in this article.
